# Sensitivity analysis of fluorescent nuclear track detectors for fast and high‐energy mono‐energetic neutron dosimetry

**DOI:** 10.1002/mp.17799

**Published:** 2025-04-20

**Authors:** Stefan Schmidt, Jeppe B. Christensen, Benjamin Lutz, Alberto Stabilini, Eduardo G. Yukihara, José Vedelago

**Affiliations:** ^1^ Department of Radiation Oncology Heidelberg University Hospital (UKHD) Heidelberg Germany; ^2^ Department of Medical Physics in Radiation Oncology German Cancer Research Center (DKFZ) Heidelberg Germany; ^3^ Heidelberg Institute for Radiation Oncology (HIRO), National Center for Radiation Research in Oncology (NCRO) Heidelberg Germany; ^4^ Medical Faculty Heidelberg Heidelberg University Heidelberg Germany; ^5^ Heidelberg Ion Beam Therapy Center (HIT) Heidelberg University Hospital Heidelberg Germany; ^6^ Department of Radiation Safety and Security Paul Scherrer Institute (PSI) Villingen PSI Switzerland; ^7^ Physikalisch‐Technische Bundesanstalt (PTB) Braunschweig Germany

**Keywords:** fast and high‐energy neutrons, fluorescent nuclear track detectors (FNTDs), neutron dosimetry

## Abstract

**Background:**

In ion beam radiotherapy, treatment radiation fields are inevitably contaminated with secondary neutrons. The energies of these neutrons can reach several hundreds of MeV. Fluorescent nuclear track detectors (FNTDs) offer a promising solution for dosimetry of fast and high‐energy neutrons, particularly given their low linear energy transfer in water (LET) detection threshold.

**Purpose:**

This study presents an experimental FNTD sensitivity analysis in six fast mono‐energetic neutron fields, comparing the response to poly allyl diglycol carbonate (PADC) neutron detectors, and investigates the feasibility of estimating ambient dose equivalent for neutrons, $H^{*}$(10). Moreover, it investigates the impact of converter thickness on the detector signal for both fast and high‐energy neutrons and analyzes the resulting differences in signal.

**Methods:**

FNTDs and PADCs were exposed to mono‐energetic neutron fields with energies of 1.2 MeV, 2.5 MeV, 5 MeV, 6.5 MeV, 14.8 MeV, and 19 MeV and evaluated based on the track density. The $H^{*}$(10) values for FNTDs were determined by applying energy calibration factors, k(E), which were determined through Monte Carlo (MC) simulations. The benchmarked MC model is employed to investigate the sensitivity of FNTDs to high‐energy neutrons up to 200 MeV for various polyethylene (PE) converter thicknesses and to analyze the detector signal, including the particle type and the recoil proton LET.

**Results:**

The sensitivity values revealed an energy dependence for FNTDs, with variations by a factor of up to 23, whereas PADC detectors showed a smaller variation, ranging from 3 to 12. Accurate $H^{*}$(10) estimation can be achieved employing MC‐derived k(E) factors, with deviations not exceeding 10%. The sensitivity values increased almost continuously up to 200MeV for PE converter thicknesses above 2mm, whereas plateaued for thinner PE converters above 10 MeV to 15 MeV. For neutrons above 20MeV, the generated fragments are deuterons, tritons and 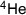, which constitutes up to 15% or more of the total fluence in a 150MeV neutron field. The recoil proton LET dropped from approximately $47\;\mathrm{keV}\;\mu m^{-1}$ to nearly one order of magnitude less between 1.2 MeV and 19 MeV, with an average LET of approximately $2\;\mathrm{keV}\;\mu m^{-1}$ at 150MeV.

**Conclusions:**

This study compares FNTD and PADC detector sensitivities, demonstrating a notable energy and converter thickness dependence for FNTDs, which is essential for precise dosimetry. Accurate $H^{*}$(10) values for fast mono‐energetic neutrons up to 19MeV were determined utilizing MC simulations. A benchmarked MC model for fast neutrons was then applied to analyze the FNTD signal for high‐energy neutrons.

## 1 INTRODUCTION

Neutron dosimetry represents a challenge within radiation protection and radiation therapy, especially considering the broad energy spectrum of secondary neutrons in proton and light ion beam therapy, with neutron energies from thermal up to high‐energy neutrons with several hundred MeV.^1^, ^2^ For clarity in this article, fast neutrons are defined as those with energies between 0.5 MeV and 20 MeV and high‐energy neutrons as those above 20MeV. The present article places particular emphasis on fast and high‐energy neutrons, in contrast to former studies that reported thermal neutron detection by employing 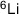 or 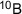 converters.^3^, ^4^ Furthermore, the out‐of‐field dose due to neutrons is dominated by fast and high‐energy neutrons, whereas the contribution of neutrons with a lower energy is in the order of a few percent.^1^, ^5^ Given that neutrons possess no charge, the only method for their detection is through the generation of secondary charged particles in a converter or radiator material that surrounds the detector.^6^

Due to the strong energy dependence of neutron cross sections, the sensitivity of neutron detectors can also strongly vary with the neutron energy, necessitating specific calibration for different neutron energies and different dosimeter configurations, including changes in converter thickness.^4^, ^7^, ^8^ Various detector types are available to determine personal and ambient dose equivalent for neutrons, $H_{p}$(10) and $H^{*}$(10). Neutron dosimeters, mainly based on thermoluminescence detectors in albedo mode and etched track detectors, have demonstrated reliable performance during neutron detector intercomparisons.^9^, ^10^ For poly allyl diglycol carbonate (PADC) detectors, the energy dependence in sensitivity has already been studied up to 100MeV, allowing them to be used in clinical settings.^7^, ^11^

Another type of neutron detector is the fluorescent nuclear track detector (FNTD), which utilizes radiophotoluminescence in a crystal to detect and store information on the interaction of ionizing radiation, for example in aluminum oxide doped with carbon and magnesium.^12^, ^13^ Like PADCs, FNTDs reveal tracks of charged particles generated by neutrons in a converter material, which is usually made of polyethylene (PE) or another material with a high hydrogen mass content.^6^, ^8^ Considering high‐energy neutrons being present in ion beam radiotherapy, FNTDs are advantageous over PADCs due to their linear energy transfer in water (LET) threshold of $0.4\;\mathrm{keV}\;\mu m^{-1}$, thereby enabling the measurement of protons with energies of up to approximately 230MeV.^12^ Consequently, FNTDs are less susceptible to sensitivity loss for high‐energy neutrons. However, most research on FNTDs in neutron dosimetry has involved broad‐spectrum radioactive sources such as 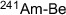, which is commonly used for neutron dosimeter calibration.^6^, ^14^ Only a limited number of studies have been conducted on the characterization of FNTDs with fast mono‐energetic neutrons.^15^, ^16^ The objective of this study is to experimentally characterize the sensitivity of FNTDs for fast mono‐energetic neutrons between 1.2 MeV and 19 MeV with different PE converter thicknesses, and compare the sensitivity values to those of PADC detectors. Furthermore, $H^{*}$(10) estimations based on Monte Carlo (MC) simulated energy calibration factors, k(E), are conducted for FNTDs. Additionally, the benchmarked MC model is used to investigate the detector signal for neutron energies up to 200MeV.

## 2 MATERIALS AND METHODS

### 2.1 Neutron irradiation facilities

The irradiations were conducted at two different facilities, namely at the Paul Scherrer Institut (PSI; Villingen, Switzerland) and at the Physikalisch‐Technische Bundesanstalt (PTB; Braunschweig, Germany). In both facilities, $H^{*}$(10) was used as quantity for dose delivery.

#### 2.1.1 Paul Scherrer Institut

The PSI operates a secondary standard reference calibration laboratory.^17^ In addition to a gamma (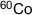, 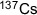) and an x‐ray irradiation facility, the lab operates an accredited secondary calibration laboratory for 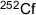, moderated 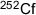 and 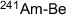. The facility is primarily utilized to calibrate and verify dose and dose rate meters. To obtain reference calibration data, FNTDs were irradiated using a 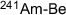 neutron source with a fluence‐averaged neutron energy of 4.05MeV and a nominal neutron emission rate of $5.206\cdot 10^{7}\;\;s^{-1}$ (reference date 04.08.2023). The source is located in a radiation room measuring 6.5m× 12.5m× 6.0m. Measurements were conducted at a distance of 28cm to the source, resulting in a dose rate of about $7991\;\mu {\mathrm{Sv}\;\;h}^{-1}$ and an expanded uncertainty of approximately 8.3% (k=2). The FNTDs were irradiated with $H^{*}$(10) values of 1 mSv, 5 mSv, 10 mSv, 15 mSv, 50 mSv and 100 mSv.

#### 2.1.2 Physikalisch‐Technische Bundesanstalt

The PTB provides reference mono‐energetic accelerator‐based neutron fields suitable for the response characterization of neutron detectors. The neutron energies available at the PTB range from 0.024 to 19 MeV. In this study, six different neutron energies, namely 1.2 MeV, 2.5 MeV, 5 MeV, 6.5 MeV, 14.8 MeV, and 19 MeV, were used in order to achieve a relatively equally spaced logarithmic distribution of the data points (see Table 1).^18^ Across all these energies, the delivered $H^{*}$(10) values ranged between 2.72 mSv and 15.3 mSv. The mono‐energetic fields are generated by proton and deuteron projectiles, accelerated with a tandetron accelerator, and bombarding tritium in a Ti(T) target or deuteron in gaseous $D_{2}$ target. In the combination of deuteron projectiles and tritium target, parasitic protons are generated due to the 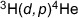 reaction, exhibiting maximum energies of 12.5MeV for 14.8MeV neutrons and 11MeV for 19MeV neutrons. To shield these protons and avoid a competing signal in the detectors, an additional 0.49mm thick aluminum sheet was placed after the target backing, not impacting the neutron and recoil proton fluence in the holder (see Table S1).

**TABLE 1:** Mono‐energetic neutron fields available at the PTB, along with their energy width and production reaction.

Neutron energy / MeV	Energy width / MeV	Production reaction	Photon contribution / %	$\phi_{\text{n, scat}}\;\cdot \;\phi_{n}^{-1}$ / %
1.2	0.168	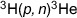	0.04	3.1
2.5	0.127	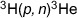	0.03	1.4
5	0.200	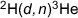	0.02	<1
6.5	0.138	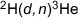	0.02	<1
14.8	0.431	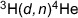	0.04	3.0
19	0.300	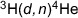	0.60	1.2

*Note*: Additionally, the relative photon contribution to $H^{*}$(10) and the ratio of neutrons scattered in the target are reported.^19^, ^20^

Abbreviation: PTB, Physikalisch‐Technische Bundesanstalt.

For the 5 MeV and 6.5MeV, measurements without the deuteron in gaseous $D_{2}$ target were conducted to quantify its background signal. In addition, a decrease in neutron fluence and a reduction in neutron energy towards the lateral sides of the irradiation field are present. Fluence correction factors are generally less than 3%, with a maximum of 3.3 % (Table S2). Given that the mean energy decrease is always less than 0.7%, no energy correction factors were applied here (Table S3).

### 2.2 Detectors

Measurements were conducted with FNTDs manufactured by Landauer Inc‐Crystal Growth Division (Stillwater, OK, USA).^12^ The detectors have dimensions of 8mm× 4mm× 0.5mm and are polished on one side to optical quality.

In addition, passive PADC track detectors were used for comparison purposes, as these detectors are already used in routine dosimetry for occupational personal monitoring.^21^ The detector material is manufactured by Track Analysis Systems Ltd. (TASL; Bristol, UK) with the trade name ${\mathrm{TASTRAK}}^{\text{TM}}$ and dimension of 20mm× 25mm× 1.5mm.

#### 2.2.1 Holder setup

**FIGURE 1:**
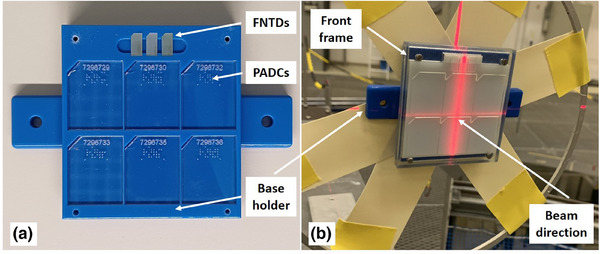
Detector holder. (a) Base holder showcasing FNTDs and PADCs in their slots. (b) Closed holder with base holder, front frame and converter sheets in irradiation position at PTB. The beam is coming from the right side parallel to the normal of the detector surface. FNTDs, fluorescent nuclear track detectors; PADCs, polyallyl diglycol carbonates, PTB, Physikalisch‐Technische Bundesanstalt.

Irradiations were conducted with 3D‐printed holders, visualized in Figure 1. The material used has the brand name VeroClear RGD810, was ordered at Stratasys (MN, USA; Product code: SDS‐06119 DE E), and has a polymerized density of about $1.19\;g\;{\mathrm{cm}}^{-3}$. The dimensions of the base holder (blue part in Figure 1) are 6.5cm× 7cm× 1cm. It has three slots for FNTDs and six slots for PADCs to account for the lower sensitivity of PADCs at the higher neutron energies used in this study.^11^ Upstream the detectors, two distinct converter sheets are employed. PE for generating recoil proton signal and polytetrafluoroethylene (PTFE) which can be used for signal correction of gammas, as FNTDs exhibit sensitivity to delta electrons.^6^ Both materials were bought from Merck KGaA (Darmstadt, Germany). Also, different thicknesses of 1 mm and 4mm were employed, with an uncertainty of ± 0.1mm. As PADC detectors possess a LET threshold of about $10\;\mathrm{keV}\;\mu m^{-1}$, rendering them insensitive to delta electrons, only FNTDs were covered with a combination of PE and PTFE, with PADCs being covered exclusively with PE.^22^ The two converter sheets are fixed with a thin 3D‐printed front frame of 1mm height (transparent part in Figure 1b). The additional signal due to the holder can be assumed to be less than 1% for the investigated neutron energies (as shown in Figure S1).

#### 2.2.2 Readout and post‐processing

##### Fluorescent nuclear track detector

A dedicated FNTD reader (FXR700RG), designed and manufactured by Landauer, was used to read the FNTDs.^23^ This confocal microscope allows for an automated readout of multiple detectors as well as for automatic surface detection. The readout was conducted at a depth of 2μm below the polished side of the crystal, consistent with previous studies.^8^, ^14^ This depth keeps the microscope's focal point within the crystal, minimizing surface‐related optical effects while preserving signal strength by limiting absorption loss. The images have a side length of 100μm, and with a readout time of 10s for 512pixels, this results in a dwell time of approximately 38.147μs per pixel. A total area of $1.0\;{\mathrm{mm}}^{2}$ below the PE converter was read for each FNTD. All readout fields were located at least 1mm distant from the edge of the detector or the PTFE converter.

For post‐processing of the microscope images, an in‐house developed MATLAB 2024b (MathWorks; Natick, Massachusetts, USA) script was utilized, presenting different image corrections as well as track spot filtering procedures (described in Section S4).^24^ Most important is the track spot filtering utilizing the principle component analysis (PCA), which yields accurate outcomes when solely examining the signal below PE.^14^ This methodology demonstrates a reasonable calibration curve for 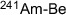, exhibiting agreement of about 10% with existing literature (Figure S4).

To accurately calculate $H^{*}$(10) values for FNTDs, information about the neutron energy must be available due to the energy‐dependence of the recoil proton yield per primary, $Y_{p}\left(E\right)$, as well as of the fluence to ambient dose conversion coefficient, $h_{\phi }\left(E\right)$. Neutron dose in terms of $H^{*}$(10) can be calculated by employing the measured track density $\rho_{t}\left(E\right)$ and k(E):

(1) $$H^{*}\left(10\right)=\frac{\rho_{t}\left(E\right)}{k(E)}$$

In this study, k(E) values were determined by employing MC simulations, where the factor $10^{9}$ is needed to present k(E) in units of ${\mathrm{mSv}}^{-1}{\mathrm{cm}}^{-2}$.

(2) $$k\left(E\right)=\frac{Y_{p}\left(E\right)\cdot 10^{9}}{h_{\phi }\left(E\right)}$$

Determined $H^{*}$(10) values are presented as normalized dose values in relation to the reference dose values. Measurement uncertainties are due to systematic uncertainties inherent to the irradiation facility and the statistical uncertainty, expressed as the standard error of the mean for the track density values of the FNTDs that were irradiated at the same time, together with uncertainties related to k(E).

##### Poly allyl diglycol carbonate

Before reading PADC detectors, developing the tracks with a chemical etching process utilizing 6.25 M NaOH is necessary.^4^ Afterwards, the detectors are neutralized and washed in preparation for further processing. Detector reading is done with a widefield microscope scanning system in combination with the TASLImage track analysis software.^25^

Two sources of uncertainty are considered: the statistical uncertainty, taken as the standard error of the mean for track density across six detectors per irradiation setting, and the systematic uncertainty from the irradiation.

### 2.3 Monte Carlo simulations

Simulations were conducted using the latest FLUktuierende KAskade (FLUKA) MC code 4.4‐1 (release date 05.07.2024), released by CERN, together with the FLUKA Advanced InteRface FLAIR 3.3‐1 (release date 17.10.2024).^26^, ^27^, ^28^ The PRECISIOn library used enables the transportation of particles down to 0.1MeV and neutrons down to $10^{-5}\;\mathrm{eV}$. For pointwise neutron transportation, JEFF‐3.3 libraries are used up to 20MeV.^29^, ^30^, ^31^ The resonance production and decay model as well as the Cascade‐Preequilibrium model PEANUT are used for high‐energy neutrons. For the simulations, calculations were spawned on five different cores, with a total of $1\cdot 10^{8}$ to $2.5\cdot 10^{9}$ events. Mono‐energetic and flat energy distributions were used for k(E) determination. The geometry was chosen following the holder used experimentally (material information can be taken from Table S4).

Detector signal simulations were conducted with the neutron energies used experimentally and the neutron energies of 66 MeV and 150 MeV, as these values are available at quasi‐monoenergetic neutron reference fields, for example at the iThemba Laboratory (iTL) for accelerator‐based sciences (Cape Town, South Africa).^20^, ^32^ For the converter thickness study, neutron energies of 1 MeV, 2 MeV, 3 MeV, 5 MeV, 7 MeV, 8 MeV, 9 MeV, 10 MeV, 15 MeV, 18 MeV, 20 MeV, 30 MeV, 50 MeV, 70 MeV, 100 MeV, 150 MeV and 200 MeV were used to cover fast and high‐energy neutrons for PE converter thicknesses of 0.5 mm, 1 mm, 1.5 mm, 2 mm, 3 mm, 4 mm and 5 mm. For the energy range between 7 MeV and 10 MeV, the spacing of the data points was reduced to better account for the change in the conversion coefficient. The simulations were conducted in a simplified geometry, excluding the holder, and for all converter thickness setups positioned adjacently. The recoil proton fluence was scored at the FNTD surface with an energy threshold of 0.4MeV to account for low‐energetic recoil protons that do not reach the scoring plane at a depth of 2μm.

Neutron and proton planar fluence (named current in FLUKA) were scored with the USRBDX estimator for uni‐ and bidirectional scoring, respectively, and compared with experimental results. The proton planar fluence was scored for all FNTDs independently and averaged over the detectors. In agreement with previous publications, detector sensitivity and $H^{*}$(10) values were calculated based on simulated k(E) values, considering proton and neutron fluence along with $h_{\phi }\left(E\right)$ values from ICRP 74.^8^, ^33^, ^34^ For $h_{\phi }\left(E\right)$ values that are not listed, Lagrangian four‐point interpolation was performed. To analyze the contribution of other ions to the signal, namely deuterons, tritons, 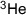, 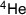, and heavier ions, fluence values were scored and normalized by the total fluence. Track‐length weighted LET was scored at 2μm depth using the USRYIELD estimator. For the binning, a width of $1\;\;\mathrm{keV}\;\mu m^{-1}$ was chosen. The average LET value was calculated by integrating the LET values.

## 3 RESULTS

### 3.1 FNTDs reveal a strong energy dependence for fast mono‐energetic neutrons

**FIGURE 2:**
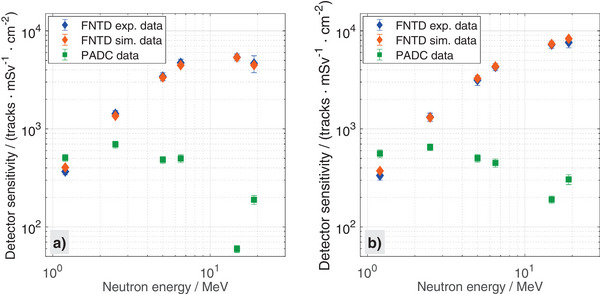
Dosimeter sensitivity investigation for FNTDs (experimentally and simulations) and PADCs (only experiments) across neutron energies ranging from 1.2 MeV to 19 MeV. Uncertainty is expressed as the standard error of the mean. Different converter thickness were employed, (a) 1mm and (b) 4mm. FNTDs, fluorescent nuclear track detectors; PADCs, poly allyl diglycol carbonates.

To investigate the sensitivity as a function of neutron energy, response values at different neutron energies were determined experimentally for FNTDs and PADCs as well as in simulations for FNTDs. For FNTDs, the experimental and simulated sensitivity values for both converter thicknesses, 1 mm and 4 mm, follow the same trend up to 6.5MeV (see Figure 2). However, for the 1mm converter and above 6.5MeV, the sensitivity first saturates and decreases again when increasing neutron energy up to 19MeV. Maximal sensitivity is about $5400\;\mathrm{tracks}\;{\mathrm{mSv}}^{-1}\;{\mathrm{cm}}^{-2}$. For the 4mm converter, however, the sensitivity is continuously increasing, reaching in the investigated energy range its maximum at 19MeV with $7600\;\mathrm{tracks}\;{\mathrm{mSv}}^{-1}\;{\mathrm{cm}}^{-2}$. For both converter thicknesses, the relationship between sensitivity and neutron energy is reproduced by simulations, with a maximum deviation from the experiments of about 12 %. The experimental sensitivity varies between neutron energies of 1.2 MeV and 19 MeV, with changes up to a factor of 14 for 1mm and 23 for 4mm. For PADC detectors, the sensitivity varies by a factor of 12 for 1mm and 3 for 4mm. Between 1.2 MeV and 2.5 MeV, sensitivity is increasing, which is followed by a decrease for higher neutron energies. At 14.8MeV, both thicknesses exhibit a strong reduction, followed by a subsequent rise at 19MeV. For the 4mm thick converter, detector sensitivity at the two highest neutron energies is increased by more than 50% compared to the 1mm values.

Overall, FNTDs revealed a strong energy dependence for fast mono‐energetic neutrons, especially compared to PADC detectors. It was observed that as the neutron energy increases, the influence of the converter thickness on the detector sensitivity becomes more pronounced.

### 3.2 Accurate $H^{*}$(10) assessment with FNTDs for fast mono‐energetic neutrons

To allow accurate $H^{*}$(10) assessment with FNTDs, energy correction has to be applied in order to account for the energy‐dependence of $h_{\phi }\left(E\right)$ and $Y_{p}\left(E\right)$. In this study, dose values for neutron energies between 1.2 MeV and 19 MeV were obtained by employing k(E) factors determined with MC simulations. The values can be taken from Table S5.

**FIGURE 3:**
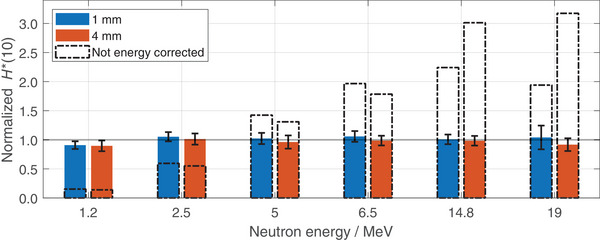
$H^{*}$(10) estimation with FNTDs in fast mono‐energetic neutron fields for two different converter thicknesses, 1 mm and 4 mm. The colored bars contain the dose values obtained with specific MC‐derived energy calibration factors normalized to the delivered dose. The dashed bars report the dose values obtained when solely applying an 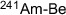 calibration factor. The uncertainties represent the total uncertainty. FNTDs, fluorescent nuclear track detectors, MC, Monte Carlo.

In Figure 3, the normalized $H^{*}$(10) values for the two different converter thicknesses are displayed in blue and orange for 1 mm and 4 mm, respectively, after energy‐specific calibration factors are applied. The dashed bars visualize the $H^{*}$(10) values if solely applying the track density to $H^{*}$(10) conversion coefficient derived from 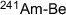 measurements. For the non‐energy corrected data at 1.2MeV, the obtained dose values underestimate the actual dose, revealing a response of about 0.1. Instead, for 2.5 MeV, 5 MeV, and 6.5 MeV, deviations are within a factor of 0.5 and 2 as the neutron energy is close to the one of 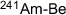. For neutron energies above, overestimation with a factor of 2 or more can be observed. For 1mm at 19MeV, the overestimation is smaller compared to 14.8MeV. When applying the energy correction factors, estimated dose values have a maximal deviation of about 10% from the reference dose value. The average deviation for each of the two converter setups is about 5%.

Overall, it has been demonstrated that $H^{*}$(10) values for fast mono‐energetic neutron fields can be accurately determined by employing k(E) factors obtained with MC simulations, resulting in a flattened detector sensitivity, thus benchmarking the MC model. Furthermore, solely applying calibration factors for 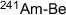 for the energy range investigated, the estimated dose values deviate by a factor of about 0.1 up to 3.2.

### 3.3 FNTD sensitivity for various converter thicknesses to fast and high‐energy neutrons

**FIGURE 4:**
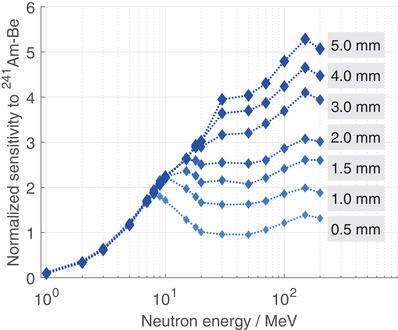
Simulated FNTD sensitivity normalized to the sensitivity for 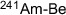 with a 1mm converter thickness for various neutron energies between 1 MeV and 200 MeV and converter thicknesses between 0.5 mm and 5.0 mm. FNTD, fluorescent nuclear track detector.

The MC model presented earlier was employed to evaluate the impact of the converter thickness on the detector sensitivity, considering more values of converter thickness and a wider neutron energy range. Figure 4 depicts the FNTD sensitivity values for recoil protons, normalized to the sensitivity with 1mm converter thickness for 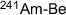. The uncertainty values are not visualized, as the mean value is about 1%, and the maximum value is less than 6%.

For all investigated converter thicknesses, the sensitivity for 1MeV amounts about 10% of that from 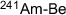 and rises to a factor of about 1.8 for 8MeV. For neutron energies above and converter thicknesses between 0.5 and 1 mm, no clear further increase in sensitivity can be observed. For a converter thickness of 1.5 mm to 2 mm, however, sensitivity rises to a factor between 2 and 3. For detectors with a converter thickness of 3mm and more, sensitivity can reach values of approximately 4 and above, with a maximum sensitivity of over 5 at 5mm and 150MeV. When the general monotonic increase in sensitivity for neutron energies below 10MeV is not taken into account, a converter thickness of 1mm results in a normalized sensitivity between 1.6 and 2.2 across the investigated energy range, while a thickness of 1.5mm results in a normalized sensitivity between 2.1 and 2.6.

It has been shown that sensitivity strongly differs with converter thickness for high‐energy neutrons, resulting in a sensitivity increase of up to 5.

### 3.4 Recoil protons are the primary contributors to the detector signal

**FIGURE 5:**
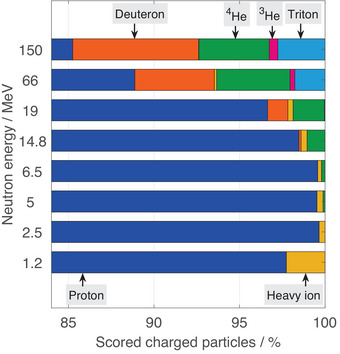
Normalized fluence contribution of recoil protons and charged fragments, in the label called scored charged particles, to the signal inside the FNTD, obtained with MC simulations. For visualization purposes, the figure only shows the upper range as protons make up at least 85% of the total fluence for all energies investigated. FNTD, fluorescent nuclear track detector; MC, Monte Carlo.

To analyze the contribution of recoil protons and fragments inside the FNTDs to the total scored charged particle fluence at different neutron energies, MC simulations were conducted. In Figure 5, the contribution of different recoil particles with an atomic number of one or greater to the total fluence is analyzed.

It needs to be mentioned that the abscissa ranges only from 84%to100% as for all investigated neutron energies, protons contribute more than 80% of the total fluence in the scoring plane. In general, for neutron energies between 1.2 MeV and 14.8 MeV, protons constitute the majority of observed charged particles with more than 97%. However, at 19MeV and higher, neutron‐induced light ions become more important. For example, 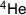 contributes almost 2% to the total fluence at 19MeV and reaches up more than 4% at 150MeV. The ratio of deuterons starts increasing from 19MeV on, making up about 7% of the charged particles at 150MeV, representing the second most contribution. A relevant contribution of triton can first be observed for 66MeV, constituting about 2%. For the remaining particle types, including 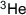 and other heavy ions, the contribution is consistently below 1% for the investigated neutron energies. However, at 1.2MeV, the relatively low recoil proton fluence results in a heavy ion contribution of about 3%. Given that recoil protons, mainly generated in elastic interactions with hydrogen, are responsible for most of the track spots in the FNTD, the following analysis will only be conducted for recoil protons.

To gain a deeper understanding of the track spot signals within the FNTD, MC simulations were performed to examine the average recoil proton energy and the average LET (see Table 2). The average proton energy is in the order of half the initial neutron energy for fast neutrons. However, for 66 MeV and 150 MeV, the average proton energy is approximately one‐third of the initial neutron energy. Considering the average LET values, one can see that the values strongly decrease with increasing neutron energy. While it is about $47\;\mathrm{keV}\;\mu m^{-1}$ at 1.2MeV, it decreases by almost one order of magnitude for 19MeV. Furthermore, the LET spectrum characteristics change for different neutron energies. While for 1.2MeV, there is a rather broad distribution with values between 25 $\mathrm{keV}\;\mu m^{-1}$ and 83 $\text{keV}\;\mu m^{-1}$, it narrows down to values between 2 $\mathrm{keV}\;\mu m^{-1}$ and 20 $\text{keV}\;\mu m^{-1}$ for 19MeV, with a most‐likely LET value in the range of 3 $\mathrm{keV}\;\mu m^{-1}$ to 4 $\mathrm{keV}\;\mu m^{-1}$, as can be taken from Figure S5.

**TABLE 2:** Average proton energy and LET for fast and high‐energy neutrons.

Neutron energy / MeV	Average proton energy / MeV	Average LET / keV·µm^−1^
1.2	0.5	47.3
2.5	1.1	29.3
5	2.3	17.8
6.5	3.0	14.6
14.8	7.5	7.0
19	9.6	5.8
66	25.6	3.0
150	47.7	1.8

*Note*: The propagated uncertainty is not specified here as the values are always 2% or less.

Abbreviation: LET, linear energy transfer in water.

**FIGURE 6:**
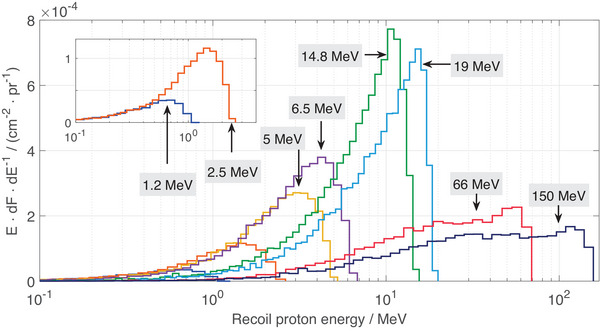
MC simulation of recoil proton fluence as a function of recoil proton energy, shown in lethargy representation. Data is recorded at a depth of 2μm in the FNTD for various initial neutron energies, with results given per primary neutron. The inset provides a magnification view for two spectra for neutron energies of 1.2 MeV and 2.5 MeV. FNTD, fluorescent nuclear track detectors; MC, Monte Carlo.

In Figure 6, the recoil proton fluence for the eight different neutron energies and a 1mm thick converter are shown against the recoil proton energy in lethargy representation. The inset in the upper left corner shows a magnification for the two smallest neutron energies. For neutron energies from 1.2 MeV to 6.5 MeV, there is a rather broad peak region, being located close to half of the neutron energy. However, for 14.8 MeV and 19 MeV, there are only narrow peaks located between half of the neutron energy and initial neutron energy. For the two high‐energy neutron energy levels investigated here, a broader distribution is observable with some small peak‐shaped region close to the initial neutron energy.

It has been shown that recoil protons contribute the most to the scored charged particle fluence for neutron energies between 1.2 MeV and 150 MeV. However, for high‐energy neutrons, neutron‐induced light particles become more important, especially deuterons, 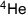 and tritons. The average LET changes strongly for fast neutrons and becomes smaller than $6\;\mathrm{keV}\;\mu m^{-1}$ for high‐energy neutrons. The proton energy spectra are also changing with neutron energy, exhibiting a pronounced peak at half of the primary neutron energy and a rather broad distribution for high‐energy neutrons.

## 4 DISCUSSION

### 4.1 Performance of FNTDs at fast mono‐energetic neutrons

In this study, the FNTD sensitivity has been experimentally investigated over a wide neutron energy range between 1.2 MeV and 19 MeV and two different converter thicknesses. It has been shown that sensitivity is strongly energy and converter thickness dependent, as sensitivity with 1mm PE starts saturating above 6.5MeV, while no saturation is observed for the 4mm in the investigated energy interval. Furthermore, these results align with previous studies where saturation was already observed above 5MeV with a 0.72mm thick PE converter.^16^

Based on this, MC simulations for various converter thicknesses and neutron energies up to 200MeV were conducted, supporting the experimental findings shown previously and predicting stronger variation in sensitivity for various converter thicknesses and for high‐energy neutrons, as shown in Figure 4. The observed increase in sensitivity is in accordance with the findings of previous studies.^8^, ^33^ The change in sensitivity can be explained by the energy‐dependent $h_{\phi }\left(E\right)$, which describes the biological effectiveness of neutrons at different energies, and the change in $Y_{p}\left(E\right)$. The inter‐dependencies of these two parameters are:

1. Neutron energy and $Y_{p}\left(E\right)$: With rising neutron energies, $Y_{p}\left(E\right)$ increases due to an increment of the range of the recoil protons, as previously reported.^33^ However, such effect manifests only when the converter has sufficient thickness, as the elastic scattering cross‐section with hydrogen decreases for increasing neutron energy.^22^
2. Equilibrium of generated and absorbed recoil protons: A neutron energy value exists where the number of generated recoil protons balances those absorbed or scattered for fixed converter thicknesses.^22^ For 1mm of PE, this is the case at about 15MeV (see Figure S6). A further increase in converter thickness would not change the sensitivity. Above this equilibrium energy, $Y_{p}\left(E\right)$ decreases due to the decrease in interaction cross‐section.^33^
3. $Y_{p}\left(E\right)$ and $h_{\phi }\left(E\right)$: For neutron energies between 1 MeV and 10 MeV, $h_{\phi }\left(E\right)$ is constant, indicating that the sensitivity is solely dependent on $Y_{p}\left(E\right)$ (see Figure S6).^34^ In the range from 10 MeV to 20 MeV, an increase in $h_{\phi }\left(E\right)$ counteracts the increase in $Y_{p}\left(E\right)$. For high‐energy neutrons, the decrease in $h_{\phi }\left(E\right)$ enhances the sensitivity and compensates for the decline in $Y_{p}\left(E\right)$ for most of the investigated converter thicknesses, leading to a moderate to strong increase in sensitivity (see Figure 4).

While FNTDs revealed a strong energy dependence of up to a factor of 23 for fast neutrons, PADCs only reveal a variation between 3 and 12, consistent with observations with radioactive sources.^4^ However, whereas PADC sensitivity variation for 4mm is concordant with previous studies, the variation with 1mm, especially for the sensitivity at 14.8MeV, reveals an increased deviation, indicating a potential presence of uncertainty associated with the detectors employed during this particular measurement run.^11^ Nevertheless, it does not affect the conclusion of the increased variation in sensitivity for FNTDs compared to PADCs.

This energy dependence is also presented when determining $H^{*}$(10) based on calibration factors derived from 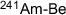 measurements. Without an energy correction, deviations in the estimated dose are in the order of 0.1 to 3.2, with only the measurements at 2.5 MeV, 5 MeV, 6.5 MeV, and 19MeV for the 1mm converter meeting the performance criteria for the energy dependence of the response according to ISO 21909‐1:2021.^35^ However, when applying MC‐derived and energy‐specific k(E) factors, the estimated dose is in good agreement for both converter thicknesses, being within a factor of 0.90 and 1.06, thus all results pass the performance criteria according to ISO 21909‐1:2021.^35^ It has to be noted that this approach is only feasible when information about the energy of the neutron field is available. Finally, while simulations were conducted with pure mono‐energetic neutron beams, the experimental neutron spectra are broader, which could contribute to the deviation observed in simulations.

### 4.2 LET value distribution

It has been shown that variation in average LET values of recoil protons is large for fast neutrons, with values of $47\;\mathrm{keV}\;\mu m^{-1}$ for 1.2MeV down to $6\;\mathrm{keV}\;\mu m^{-1}$ for 19MeV and $1.8\;\mathrm{keV}\;\mu m^{-1}$ for 150MeV. These values are found to be consistent with those observed in the program ATomic Interaction with MAtter (ATIMA), revealing an energy loss of about $23\;\mathrm{keV}\;\mu m^{-1}$ for 1.2MeV protons, compared to the lowest observed LET value of $25\;\mathrm{keV}\;\mu m^{-1}$ here (see Figure S5).^36^ A comparison of the simulated values with those from an analytical model shows higher LET values in the simulations.^37^ For 1.2MeV neutrons, the model predicts LET values of $33\;\mathrm{keV}\;\mu m^{-1}$. The deviations may result from the assumption that the recoil proton energy is half of the initial neutron energy.

It should also be noted that, especially for the high‐energy neutrons, the simulated LET values might underestimate the actual LET since the values were determined only for protons and not for other charged fragments.

### 4.3 Optimal converter thickness for neutron dosimetry

In practical terms, the question arises as to which type of converter should be employed in neutron dosimetry. It is, therefore, necessary to determine whether the maximum sensitivity for each neutron energy is always beneficial, or whether the focus should be more on a uniform sensitivity, which can be better achieved with a thinner converter. In a previous study, the change of $h_{\phi }\left(E\right)$ was modeled between thermal and fast neutrons by the detector sensitivity, utilizing different converter materials and thicknesses.^38^ In this study, the focus was on fast and high‐energy neutrons. It was demonstrated that utilizing a converter thickness of 1mm results in a normalized sensitivity between 1.6 and 2.2 for neutron energies within the range of 10 MeV to 200 MeV suggesting the feasibility of reasonable dose estimation with only a single conversion coefficient (see Figure 4). Nevertheless, if $H^{*}$(10) is to be measured solely for a specific neutron energy, selecting a thicker converter may prove more practical, as it leads to enhanced sensitivity and enables the lowering of detection limits.

In this study, the MC model was benchmarked with fast neutrons up to 19MeV, revealing a good agreement. However, in FLUKA, neutron interactions are treated differently for fast and high‐energy neutrons. Therefore, further experimental validation of the model for neutron energies above 20MeV would be preferable. However, benchmarking MC models with high‐energy neutrons is hampered by the fact that only a limited number of neutron facilities are available that provide high‐energy neutrons that are — ideally — quasi‐monoenergetic.^39^ To address this challenge, it would be beneficial to conduct MC simulations with different MC codes and nuclear libraries to improve the reliability of the results. Previous studies have revealed that there can be strong differences when simulating neutrons using different MC codes.^40^, ^41^

### 4.4 Implication for neutron dosimetry with FNTDs in ion beam radiotherapy

Due to the strong energy dependence of the FNTD sensitivity and the change in signal for high‐energy neutrons, further research is required to explore the potential applications of FNTDs for neutron dosimetry in ion beam radiotherapy, where high‐energy neutrons with energies up to hundreds of MeV are generated. Thus it is recommended to characterize FNTDs across the full neutron energy range in ion beam radiotherapy, as done for PADC detector.^11^ This should also contain a more detailed analysis of the angular response of the dosimeter, particularly in the context of measuring $H^{*}$(10). Although the method demonstrated in this study worked for mono‐energetic neutron fields, this may not be the case for the broad secondary neutron fields present in ion beam radiotherapy. Additionally, further analysis of the track signal is required as the signal observed in the detector becomes more diverse for increased neutron energies (see Figure 6). For high‐energy neutrons, LET values decrease, rendering the application of general PCA for signal discrimination, as used in this study, more challenging. Advanced techniques such as 3D particle tracking could offer a solution.^42^

Besides, when measuring in ion beam radiotherapy, the fragmentation of ions, which creates a competing signal in addition to the actual neutron‐induced signal, must also be taken into account. One potential solution is to modify the $H^{*}$(10) determination procedure from a track‐density based to a LET‐based approach, as already done for PADC detectors with encouraging outcomes.^43^

## 5 CONCLUSION

This article presents a sensitivity analysis for Fluorescent Nuclear Track Detectors (FNTD) and poly allyl diglycol carbonate (PADC) detectors for fast mono‐energetic neutrons between 1.2 MeV and 19 MeV and two different converter thicknesses. It has been demonstrated that FNTDs exhibit a stronger energy dependence than PADCs, which must be taken into account when conducting dosimetry. Ambient dose equivalent values for neutrons, $H^{*}$(10), are estimated by employing energy calibration factors, k(E), determined through Monte Carlo (MC) simulations, reporting all dose values to be within 0.90 and 1.06 of the reference values and thus passing the energy response criteria from ISO 21909‐1:2021. Given this strong agreement between results of MC simulations and experiments for fast mono‐energetic neutrons, simulations for high‐energy neutrons were conducted, reporting an increase in sensitivity of a factor of 5 between the value at 1mm for 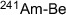 and at 5mm for 150MeV. Nevertheless, a converter thickness of 1mm yields a more uniform sensitivity across the investigated energy range, thereby reducing the energy dependence. Furthermore, a detailed analysis was conducted on the characteristics of the detector signal for both fast and high‐energy neutrons. In the case of fast neutrons, the signal is comprised predominantly of recoil protons, representing over 95% of the total signal. However, for high‐energy neutrons of 66 MeV and 150 MeV, a notable contribution from deuterons, 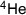, and triton can be anticipated. Moreover, it was found that the linear energy transfer in water (LET) for neutron energies between 1.2 MeV and 19 MeV decreased by an order of magnitude. For high‐energy neutrons, the average LET values are observed to be less than $6\;\mathrm{keV}\;\mu m^{-1}$, which presents an important challenge for the detection of tracks.

## CONFLICT OF INTEREST STATEMENT

The authors declare no conflicts of interest.

## Supporting information

Supporting Information
