# Supplementary material for: Sensitivity analysis of fluorescent nuclear track detectors for fast and high‐energy mono‐energetic neutron dosimetry
Source: Med Phys. 2025 Apr 20;52(7):e17799. doi: 10.1002/mp.17799 (PMC12257915; doi:10.1002/mp.17799)
Supplement: Supplementary file 1 — Supporting Information [file MP-52-0-s001.pdf]

Supplementary Material

Sensitivity analysis of fluorescent nuclear track detectors for fast and high-energy mono-energetic neutron dosimetry

Stefan Schmidt[1,2,3,4,5], Jeppe B. Christensen[6], Benjamin Lutz[7], Alberto Stabilini[6], Eduardo G. Yukihara[6], José Vedelago[1,2,3]

[1]Department of Radiation Oncology, Heidelberg University Hospital (UKHD), Heidelberg, Germany; [2]Department of Medical Physics in Radiation Oncology, German Cancer Research Center (DKFZ), Heidelberg, Germany; [3]Heidelberg Institute for Radiation Oncology (HIRO), National Center for Radiation Research in Oncology (NCRO), Heidelberg, Germany; [4]Medical Faculty Heidelberg, Heidelberg University, Heidelberg, Germany; [5]Heidelberg Ion Beam Therapy Center (HIT), Heidelberg University Hospital, Heidelberg, Germany; [6]Department of Radiation Safety and Security, Paul Scherrer Institute (PSI); [7]Physikalisch-Technische Bundesanstalt (PTB), Braunschweig, Germany;

Contents

S1 Aluminum shielding for parasitic protons 2

S2 Fluence and energy reduction for mono-energetic neutron measurements 3

S3 Analysis of the holder design and its impact on the results 5

S4 Image post-processing 7

S5 Post-processing validation with ^{241}Am-Be measurements 10

S6 Materials used in the Monte Carlo simulations 11

S7 Energy calibration factors 12

S8 Linear energy transfer distribution 13

S9 Recoil proton yield for different neutron energies 14

References 15

S1 Aluminum shielding for parasitic protons

To estimate the impact of parasitic protons and select appropriate shielding for the 14.8 MeV and 19 MeV neutron beams, Monte Carlo (MC) simulations were conducted to evaluate changes in recoil proton and neutron fluence insight the detector. Aluminum was chosen as a shielding material. For the required aluminum thickness estimation, the intrinsic shielding capability of the polyethylene (PE) converter is considered as well.

An initial estimation of the required aluminum thickness was performed using the program ATomic Interaction with MAtter (ATIMA), based on the maximum proton energy of 12.5 MeV and a minimum PE thickness of 0.9 mm to account for manufacturing uncertainties[1]. An aluminum thickness of 0.45 mm together with the PE converter does fully absorb parasitic protons, so the next available thickness, 0.49 mm, was selected. This estimate is conservative, as the parasitic protons already lose energy between the source and detector. Table S1 presents the changes in neutron and recoil proton fluence with and without the additional aluminum shielding. For the two investigated neutron energies, both recoil proton and neutron fluence remained unchanged with the aluminum shielding.

Table S1: Impact of 0.49 mm aluminum shielding to recoil proton and neutron fluence in the holder for different neutron energies. Ratio represents fluence with shielding over fluence without shielding. Uncertainty values are below 0.1 % and therefore not presented here.

Neutron energy / MeV	14.8	19
Proton fluence change / %	-0.03	0.02
Neutron fluence change / %	< 0.01	< 0.01

S2 Fluence and energy reduction for mono-energetic neutron measurements

For the measurements conducted at the Physikalisch-Technische Bundesanstalt (PTB; Braunschweig, Germany), the holder was positioned in the isocenter, as indicated by the room laser. However, the actual positions of the detectors inside the holder deviated from the isocenter, leading to reductions in recorded fluence and energy values. The change in fluence due to lateral shifts from the isocenter is influenced primarily by the distance between the source and the holder, following the inverse-square law.

The reduction in energy at lateral positions is affected by the initial projectile momentum and the distance. The angular dependence of the emitted neutron increases with the initial projectile momentum. For instance, the momentum of deuterons for neutrons of 14.8 MeV is approximately an order of magnitude smaller than that of 19 MeV neutrons. This results in a quasi-uniform distribution of neutrons at 14.8 MeV, while the angular dependence for 19 MeV neutrons is significantly higher due to the increased energy.

Table S2 shows the changes in neutron fluence, while Table S3 presents the changes in neutron energy resulting from lateral shifts from the isocenter for Fluorescent Nuclear Track Detectors (FNTD) and poly allyl diglycol carbonate (PADC) detectors[2]. Detectors located in line with the vertical laser are labeled 'center,' while those with an additional lateral shift are labeled 'lateral'. Due to the symmetry of the system, two distinct correction factors are sufficient for each detector type, corresponding to the specific energies. Maximal position shifts from the isocenter are up to 2 cm horizontally and 3 cm vertically.

In this study, only the reduction in fluence is addressed by correcting the track density accordingly. Energy reduction is not considered, as the values fall below the full width at half maximum (FWHM) of the main neutron peak and thus have minimal impact on the results.

Table S2: Fluence reduction in relative values for FNTD and PADC detectors for the six different mono-energetic neutron energies used in this study. Values are for the two different positions with respect to the isocenter, 'center' position with only a vertical shift and 'lateral' with a combined vertical and horizontal shift.

	FNTD		PADC	
Neutron energy / MeV	center / %	lateral / %	center / %	lateral / %
1.2	-0.5	-0.5	-0.2	-0.5
2.5	-0.6	-0.6	-0.2	-0.7
5	-0.7	-0.7	-0.4	-1.1
6.5	-0.9	-1.0	-0.5	-1.7
14.8	-0.4	-0.4	-0.1	-0.3
19	-3.1	-3.2	-1.2	-3.3

Table S3: Energy reduction in relative values for FNTD and PADC detectors for the six different mono-energetic neutron energies used in this study. Values are for the two different positions with respect to the isocenter, 'center' position with only a vertical shift and 'lateral' with a combined vertical and horizontal shift.

	FNTD		PADC	
Neutron energy / MeV	center / %	lateral / %	center / %	lateral / %
1.2	-0.5	-0.5	0.0	-0.1
2.5	-0.4	-0.4	0.0	-0.1
5	-0.3	-0.3	0.0	-0.1
6.5	-0.3	-0.3	0.0	-0.1
14.8	0.0	0.0	0.0	-0.0
19	-0.7	-0.7	0.0	-0.3

S3 Analysis of the holder design and its impact on the results

To identify an optimal holder design, various design elements were analyzed using MC simulations. Recoil proton fluence values were compared to those without a holder. Simulations were performed for neutron energies of 1.2 MeV and 19 MeV. The following cases were investigated:

- Case 1 - Initial holder: Original geometry, entrance window size matching the detector size and a front frame height of 2 mm.
- Case 2 - Frame height: Reduced frame height to 1 mm.
- Case 3 - Scoring margin: Additional scoring margin of 1 mm on each side of the detector.
- Case 4 - Entrance window size: Window size increased by 0.5 mm on each side.
- Case 5 - Final holder design: Combination of all three design improvements.

Figure S1 shows the relative deviation in scored proton fluence for the five different cases compared to the no-holder scenario. At 1.2 MeV, all cases resulted in a similar signal increase of 0.5 % or less. No relevant signal increase was observed for any case, as the signal being created in the holder can not reach the scoring plane due to the low energy of the recoils. At 19 MeV, the initial holder (case 1) showed an energy-dependent signal increase of about 7 %. Adjusting the holder in cases 2 and 3 reduced the additional signal to (3.2 ± 0.4) % and (3.0 ± 0.4) %, respectively, while cases 4 limited the additional signal to (2.0 ± 0.3) %. Combining all improvements (case 5) resulted in an additional signal by the holder of less than 1 %.

This demonstrates that the design elements can influence the recoil fluence, particularly at higher neutron energies. However, it has been shown that the final holder design has only a neglectable impact on the track density, allowing for energy-independent measurements between 1.2 MeV to 19 MeV.

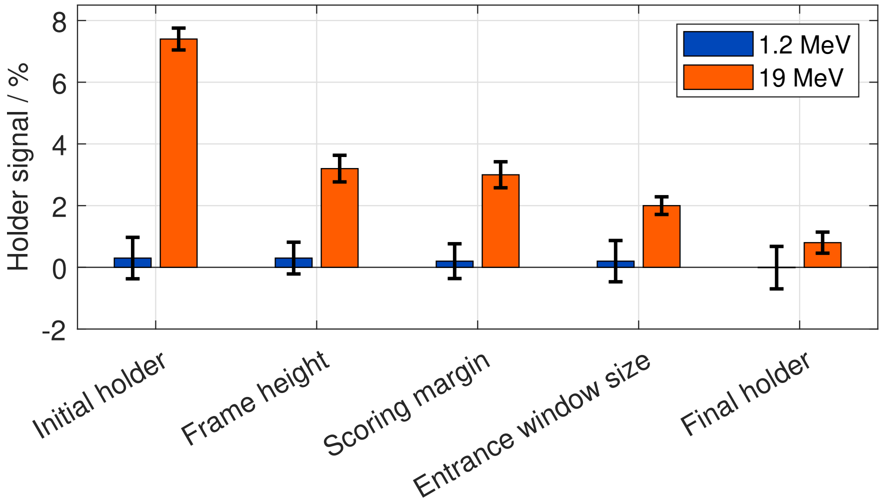

Figure S1: Analysis of additional recoil proton fluence generated in the holder for five different cases and for two different neutron energies.

S4 Image post-processing

Image post-processing of the microscope images is performed using MATLAB 2024b (MathWorks, Natick, MA, USA)[3]. Input files include two microscope images (fluorescence and reflection) in *.png* format and a text configuration file. The configuration file contains parameters like field size, imaging time, and the number of readout fields, enabling automated processing across multiple fields.

In the first post-processing step, a reflection check is performed to detect surface contamination or crystal heterogeneities using the reflection image. A Sobel kernel identifies edges, which are then used to mask corresponding pixels in the fluorescence image, reducing false positive track spots. The masked area is considered in the final track density calculation. Background correction follows the method presented by Stabilini et al. (2020), combining rolling ball correction (radius: 30 pixels) and image smoothing with a Gaussian filter (filter size: 3, sigma: 2)[4,5]. For image segmentation, different approaches were employed in the past[5,6]. One promising approach is the maximum entropy method that maximizes the entropy of the pixel intensity distribution in the image[7]. Foreground entropy peaks at the threshold level where the signal in the image can be best segmented, as shown in Figure S2. In low-signal fields (Figure S3), the absence of a clear foreground peak makes threshold determination challenging.

This led to the implementation of a new segmentation procedure effective even in low-signal readout fields. The method works by progressively changing the threshold value to identify small clusters of connected pixels that surpass the threshold, corresponding to background pixels starting to be segmented. Two parameters must be defined for this technique: (1) the size of a small cluster, here a value of less than three connected pixels is chosen, and (2) a threshold frequency for small clusters. When the frequency exceeds this threshold (set to 5 % of the maximum change), the corresponding intensity value is selected as the segmentation threshold.

The central images in Figure S2 and Figure S3 illustrate this process. The orange curve represents the frequency of small clusters (the first derivative of their count at each intensity). In high-signal samples, the identified threshold closely matches that of the maximum entropy method. The green areas in the right plots show regions segmented by both methods, while

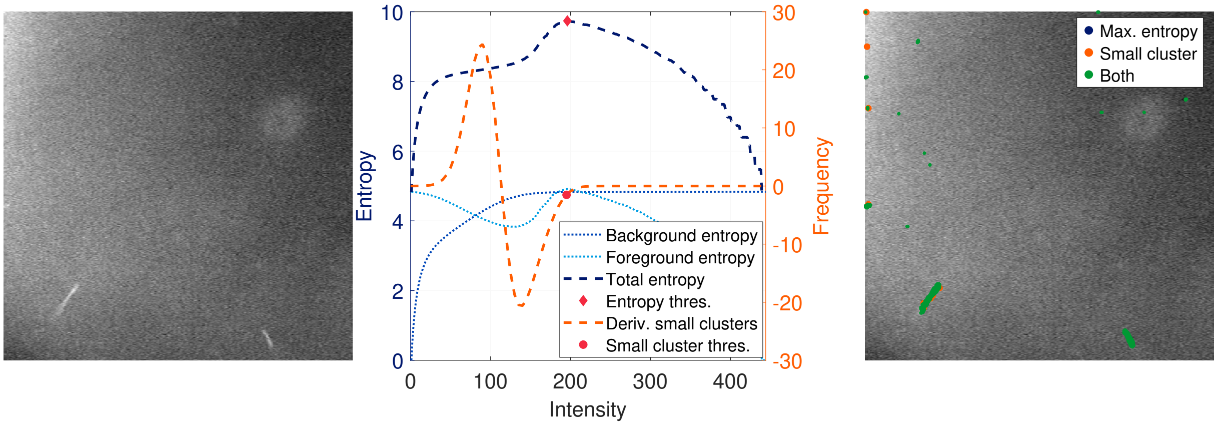

Figure S2: Comparison of maximum entropy and small clustering thresholding for a high-signal FNTD sample irradiated with 1 mSv using an ^{241}Am-Be source. The raw microscope image is shown on the left, with both segmentation methods visualized in the middle, and the final segmented image on the right.

orange and blue areas correspond to pixels segmented only by the small cluster or the maximum entropy method, respectively. The small cluster method sets a slightly lower threshold, resulting in a few additional pixels being segmented, which does not affect the overall results due to their small size. In low-signal samples (Figure S3), the small cluster method identifies only a limited number of pixels (shown in orange or green), whereas the maximum entropy method segments a larger portion of the image due to an incorrectly selected threshold value. This demonstrates the efficiency of the small cluster method for low-signal samples, revealing its advantages over the maximum entropy technique.

After identifying the threshold value, a binary matrix is applied to the image to determine properties such as area, maximum and mean intensity, and centroid of each spot. Morphological filtering then excludes clusters with less than 30 pixels or more than 500 pixels. To resolve merged track spots, a cluster identification process is used, analyzing intensity distribution, circularity, convex hull, and azimuthal angle of the potential sub-spots. Both, delta electron spots and recoil proton spots are segmented if their intensity exceeds the threshold. To isolate the recoil protons signal caused by neutrons, one method is to use different converter materials and subtract the track densities. For example, polytetrafluoroethylene (PTFE) generates only delta electron tracks, while PE produces both delta electron and recoil proton tracks. By subtracting the PTFE track density from that of PE, the neutron-

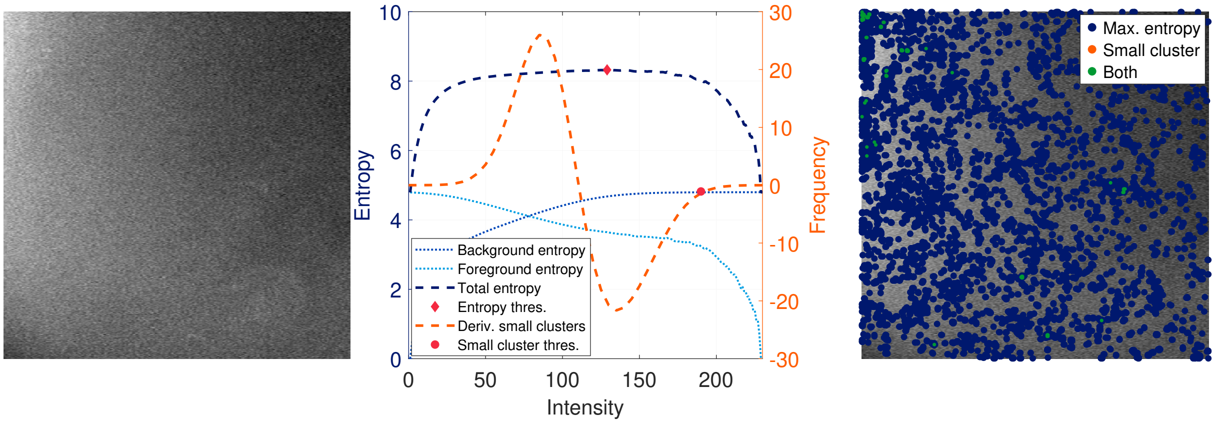

Figure S3: Comparison of maximum entropy and small clustering thresholding for a low signal sample of a FNTD irradiated with 1 mSv with an ^{241}Am-Be source. The raw microscope image is shown on the left, with both segmentation methods visualized in the middle, and the final segmented image on the right.

induced signal can be extracted[8]. Alternatively, the principal component analysis (PCA) can be employed where different properties of the track spot are condensed to reduce the dimensionality of track spot data while preserving variability. Analyzing the principal components allows differentiation between gamma-ray and neutron-induced signal, improving accuracy during data post-processing and allowing to only analyze the signal below the PE converter[9].

S5 Post-processing validation with ^{241}Am-Be measurements

To validate the post-processing algorithm used in this study and to allow comparison to other research, measurements with a reference ^{241}Am-Be source at the Paul Scherrer Institute (PSI; Villingen, Switzerland) were conducted and a calibration curve was established for dose levels of 1 mSv, 5 mSv, 10 mSv, 15 mSv, 50 mSv and 100 mSv. The resulting curve is presented in Figure S4.

For the linear fit, the standard error of the mean for each dose value is used as weights. The horizontal error bars are derived from the systematic uncertainties provided by PSI. The coefficient of determination is 0.999, indicating that the experimental outcomes can be well-modeled by the following linear fit: #tracks = (2400 ± 20) x $H^*(10)$ mSv^{-1} cm^{-2}. The slope of the calibration curve is in good agreement with values that can be found in literature, with deviations of about 10 %[6,10,11].

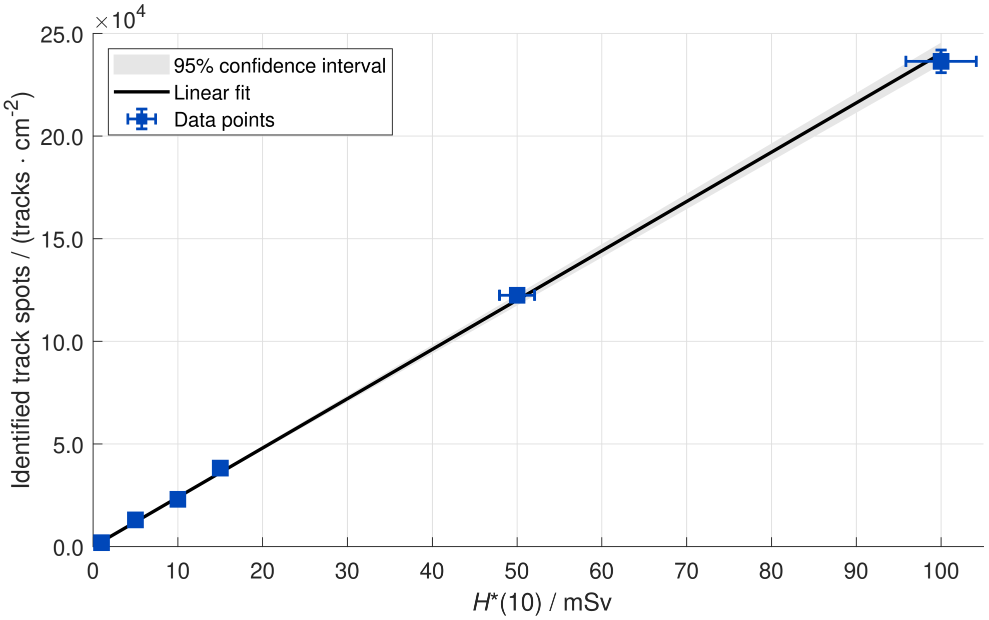

Figure S4: Calibration curve for samples irradiated with an ^{241}Am-Be source, evaluated with the post-processing procedure described previously. A weighted linear fit results in #tracks = (2400 ± 20) x $H^*(10)$ mSv^{-1} cm^{-2} with R^2 =0.999.

S6 Materials used in the Monte Carlo simulations

Table S4 lists information about the materials used in the MC study. For the 3D printed holder, a plexiglas acrylic composition of $C_5H_8O_2$ was assumed, as precise values are not available.

Table S4: Material specifications used in the MC simulations. For air, a pre-defined compound for dry air at NTP conditions was used.

Material name	Molecular formula	Density / $(g \cdot cm^{-3})$
Air	pre-defined	pre-defined
Aluminum oxide	Al_2O_3	3.97
Polyethylene (PE)	C_2H_4	0.95
Plexiglas acrylic	$C_5H_8O_2$	1.19
Poly allyl diglycol carbonate (PADC)	$C_{12}H_{18}O_7$	1.3
Polytetrafluoroethylene (PTFE)	C_2F_4	2.2

S7 Energy calibration factors

Table S5 displays the energy calibration factors k(E) for the 1 mm and 4 mm converter. These factors can be calculated according to Equation 2 and are used to estimate the detector sensitivity values in Figure 2.

Table S5: MC-derived energy calibration factors, k(E), for a 1 mm and 4 mm thick polyethylene converter, used to determine neutron ambient dose equivalent based on the measured recoil proton fluence. The values were obtained for neutron irradiation, which impinged perpendicular to the surface.

	k(E) / ($mSv^{-1} \cdot cm^{-2}$)	
Energy / MeV	1 mm	4 mm
1.2	404 ± 3	373 ± 2
2.5	1356 ± 9	1305 ± 3
5	3341 ± 11	3268 ± 10
6.5	4462 ± 17	4343 ± 25
14.8	5342 ± 19	7349 ± 26
19	4476 ± 10	8312 ± 36

S8 Linear energy transfer distribution

In Figure S5, the linear energy transfer in water (LET) distribution of recoil protons originating from 1.2 MeV and 19 MeV neutrons are visualized. While the distribution for 1.2 MeV neutrons is rather broad with LET values mainly between 25 keV μm^{-1} to 83 keV μm^{-1} and an average value of about 47 keV μm^{-1}, the distribution is rather sharp for 19 MeV with LET values between 2 keV μm^{-1} to 20 keV μm^{-1} and an average LET value of 6 keV μm^{-1}. This visualizes the difficulty of measuring fast and high-energy neutrons, as LET values become rather small and track spot intensities decrease.

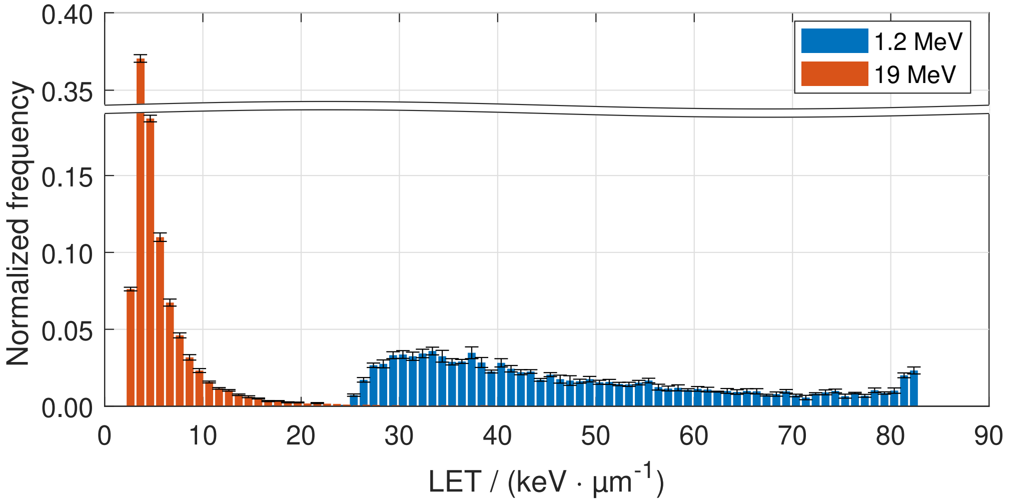

Figure S5: Visualization of LET distribution for different neutron energies. The axis is broken between 0.19 and 0.34.

S9 Recoil proton yield for different neutron energies

Figure S6 illustrates the recoil proton yield per primary neutron (Y_p) for MC simulations as a function of neutron energy for two different converter thicknesses, namely 1 mm and 4 mm. The inverse of the fluence-to-ambient dose conversion coefficient, $h_\phi(E)$, is plotted on the right y-axis, in order to highlight the two counteracting tendencies impacting the sensitivity value, as per Equation 2[12].

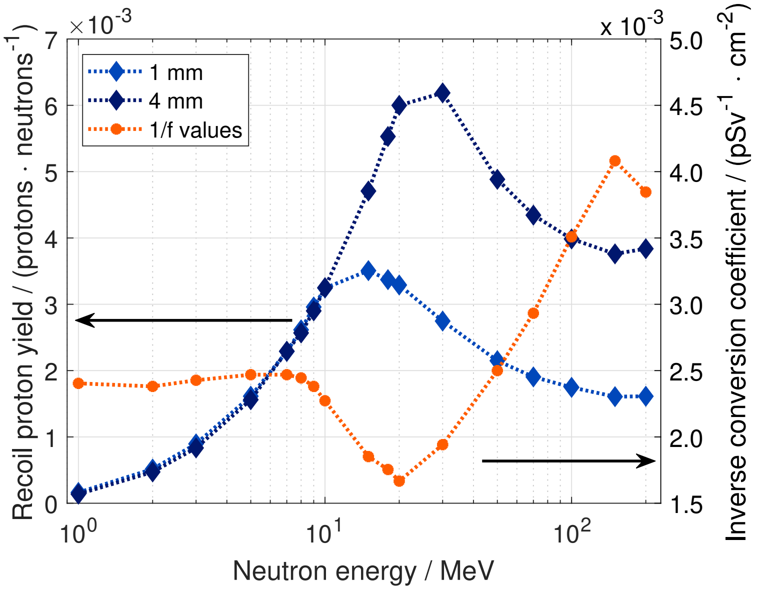

Figure S6: Recoil proton yield and inverse fluence to ambient dose conversion coefficient for two different converter thicknesses, namely 1 mm and 4 mm.

References

[1] Geisel H, Scheidenberger C, Malzacher P, Kunzendorf J, Weick H. WebAtima - Energy Loss Calculator, Accessed August 8, 2024.

[2] Drosg M, DROSG-2000: Neutron source reactions. Data files with computer codes for 56 monoenergetic neutron source reactions, *IAEA*, 2000.

[3] The MathWorks Inc., MATLAB version: 24.2.0 (R2024b), Natick, Massachusetts: The MathWorks Inc. https://www.mathworks.com.

[4] Hanson AJ. THE ROLLING BALL. *Graphics Gems III (IBM Version)* 1992;51–60. doi:10.1016/B978-0-08-050755-2.50023-3.

[5] Stabilini A, Akselrod MS, Fomenko V, Greilich S, Harrison J, Yukihara EG. 3D track reconstruction of neutron-induced recoil protons in fluorescent nuclear track detectors (FNTDs). *Radiat. Meas.* 2020;137:106438. doi:10.1016/j.radmeas.2020.106438.

[6] Schmidt S, Stabilini A, Thai LYJ, Yukihara EG, Jäkel O, Vedelago J. Converter thickness optimisation using Monte Carlo simulations of Fluorescent Nuclear Track Detectors for neutron dosimetry. *Radiat. Meas.* 2024;173:107097. doi:10.1016/j.radmeas.2024.107097.

[7] Kapur J, Sahoo P, Wong A, A new method for gray-level picture thresholding using the entropy of the histogram. *Lect. Notes Comput. Sc.* 1985;29(3):273–285. doi:10.1016/0734-189X(85)90125-2.

[8] Sykora GJ, Akselrod MS. Novel fluorescent nuclear track detector technology for mixed neutron-gamma fields. *Radiat. Meas.* 2010;45(3–6):594–598. doi:10.1016/j.radmeas.2010.01.037.

[9] Stabilini A, Akselrod MS, Fomenko V, Harrison J, Yukihara EG. Principal Component Analysis applied to neutron dosimetry based on PADC detectors and FNTDs. *Radiat. Meas.* 2021;141:106516. doi:10.1016/j.radmeas.2021.106516.

[10] Akselrod GM, Akselrod MS, Benton ER, Yasuda N. A novel Al_2O_3 fluorescent nuclear track detector for heavy charged particles and neutrons. *Nucl. Instrum. Meth. B.* 2006;247(2):295–306. doi:10.1016/j.nimb.2006.01.056.

[11] Stabilini A, Kiselev D, Akselrod MS, Yukihara EG. A Monte-Carlo study on the fluorescent nuclear track detector (FNTD) response to fast neutrons: Which information can be obtained by single layer and 3D track reconstruction analyses? *Radiat. Meas.* 2021;145:106609. doi:10.1016/j.radmeas.2021.106609.

[12] ICRP, 1996, Conversion Coefficients for use in Radiological Protection against External Radiation. ICRP Publication 74. Ann. ICRP 26 (3–4).
